# Developing mental health care of LGBTIQ+ people in Croatia- research review

**DOI:** 10.1192/j.eurpsy.2023.2397

**Published:** 2023-07-19

**Authors:** I. Zegura

**Affiliations:** Department for psychotic disorders, University Psychiatric Hospital Vrapče, Zagreb, Croatia

## Abstract

**Introduction:**

Despite the general awareness of necessity for implementing development of competences in working with LGBTIQ+ people as the obligatory course within the education of mental health professionals, it is still not the educational standard in Croatia.

**Objectives:**

Within the perspective of the past 20 years, the development of educational programs aiming to develop LGBTIQ+ affimrative and informed practice in the field of mental health care, togeather with the review of the most crucial research results in the field of LGBTIQ+ mental health will be given.

**Methods:**

The results obtained from several research on national samples of LGBTIQ+ people, psychology students and psychologists are analysed using mainly qualitative methodology and to the small extent quantitative methodology.

**Results:**

Psychologists are perceived as a profession that respects diversity and actively reduces stereotypes and prejudice but there are still some obstacles to overcome within the profession. In traditional cultures appreciation of human rights of LGBT people is unfortunately not a guiding principle. Research on Croatian university students showed that slightly positive attitudes toward LGBT people were stable and remained unchanged from the 2005 till 2013. Specific attitudes toward human rights of lesbians and gays are ranging from moderately negative to moderately positive. The most frequent forms of discrimination and/or violating human rights of LGBT people are: usage of offensive, humiliating, pejorative and oppressive language, ignoring of LGBT people, attentional exclusion of LGBT people, threatening with physical violence. Stress resilience, social support, and inclusion in the LGBT society are key determinants of different indicators of mental health in this population. Although 64.4 % of transgender participants are highly informed about the new legalization, only 24.5 % are very satisfied with it. Trans-women, in comparison to trans-men, have significantly lower levels of quality of life and experience significantly higher levels of sexual violence. Based on the online research among the professionals from the Croatian national list of experts in thefield of health care of trans people the percieved level of transphobia in sociaty is abowe the average, as well as percieved impact of the COVID- 19 pandemic on acessibility and slowing down of standard diagnostic procedures.

**Conclusions:**

Significant political and social change for sexual and gender minority people in Croatia in the last several years have contributed to a greater public visibility of LGBTIQ+ people. As allies of LGBTIQ+ people and even as belonging to the LGBTIQ+ population, psychologists, psychiatrists and other mental health professionals have the leading role in ensuring that the results of their scientific findings and professional corpus of knowledge have ethical and practical implementation and beneficial impact on society.

**Disclosure of Interest:**

None Declared

